# Development and Application of a High-Resolution Melting Analysis with Unlabeled Probes for the Screening of Short-Tailed Sheep *TBXT* Heterozygotes

**DOI:** 10.3390/ani12060792

**Published:** 2022-03-21

**Authors:** Guang Yang, Caiyun Wang, Hong Su, Daqing Wang, Aolie Dou, Lu Chen, Teng Ma, Moning Liu, Jie Su, Xiaojing Xu, Yanyan Yang, Tingyi He, Xihe Li, Yongli Song, Guifang Cao

**Affiliations:** 1 School of Life Sciences, Inner Mongolia University, Hohhot 010021, China; yangguangimu@163.com (G.Y.); lixh@imu.edu.cn (X.L.); 2 School of Veterinary Medicine, Inner Mongolia Agricultural University, Hohhot 010010, China; wangcaiyun688@163.com (C.W.); hongsu1995@126.com (H.S.); wangdaqing050789@126.com (D.W.); douaolei@126.com (A.D.); chenlu736@163.com (L.C.); mateng7330@126.com (T.M.); momo4237@126.com (M.L.); 13847172449@139.com (J.S.); 15162265618@163.com (X.X.); 3 Institute of Animal Husbandry, Inner Mongolia Academy of Agricultural and Animal Husbandry Sciences, Hohhot 010031, China; swallow_0088@163.com (Y.Y.); yier.goal@163.com (T.H.)

**Keywords:** short-tailed trait, sheep, *TBXT*, HRM

## Abstract

**Simple Summary:**

*TBXT* (c.333G > C; c.334G > T) has been identified as a molecular genetic marker in short-tailed sheep. This paper describes a high-resolution melting (HRM) analysis using unlabeled probes and asymmetric PCR for the detection of genetic variants of *TBXT* in short-tailed sheep populations. The detection results of this method are consistent with those of Sanger sequencing and can help farmers with marker-assisted breeding.

**Abstract:**

The short-tailed phenotype has long been considered one of the best traits for population genetic improvement in sheep breeding. In short-tailed sheep, not only is tail fat eliminated but also the pubic area is exposed due to the lack of a tail covering, giving them an advantage in reproduction. Recent studies have shown that two linked mutations in sheep *TBXT* at nucleotides 333 and 334 are associated with the short-tailed phenotype. In the population of short-tailed sheep, several heterozygous mutants of this gene are found. In our research, we used high-resolution melting (HRM) to identify homozygous and heterozygous genotypes in a flock of short-tailed sheep and compared the results with those of Sanger sequencing, which were identical. This demonstrates that our established HRM method, a rapid and inexpensive genotyping method, can be used to identify homozygous and heterozygous individuals in short-tailed sheep flocks.

## 1. Introduction

The tails of most animals serve multiple functions in nature [1], including balance, movement, information exchange with peers [1,2], and swatting flies to remove them from the animals. After a long period of domestication, sheep with different tail length phenotypes have been developed [3,4], including the fat-long-tail, fat-short-tail, fat-waist-tail, slender tail, and thin-short-tail phenotypes. There is evidence [5,6] that the ancestors of sheep had a narrow tail phenotype that evolved into a fat-tail phenotype as they adapted to their frigid living environment. Sheep in China can be roughly divided according to their tail phenotypes into fat-waist-tail and thin-short-tail [3,7]. In several areas [8], long-tailed sheep are the main source of the fat intake of the local population but this phenotype presents many disadvantages [9], including that the overly long tail covers the pubic area, which makes mating inconvenient, and sheep with fat tails require more feed [10], which increases the cost of rearing. Although the tail docking of fat-tailed sheep is an option [11], this not only increases the labor of the farmer but is also exceedingly painful for the sheep; therefore, this is detrimental in terms of animal welfare [12,13] and, as a result, tail docking is controversial [11,13,14]. Short-tailed sheep accumulate more intramuscular fat than fat-tailed sheep, leading to more tender meat [14]. As a result, short-tailed sheep are more popular with buyers and producers are more likely to breed sheep with short tails [9,15,16].

Genes potentially associated with the formation of the short-tail phenotype in sheep have been found in many studies such as bone morphogenetic protein 2 (*BMP2*) [7,17], platelet-derived growth factor D (*PDGFD*) [16,17] and T-box transcription factor T (*TBXT*) [18,19], among which the mutation of *TBXT* is presumed to be related to the number of tail vertebrae. In a 1990 study [20], it was discovered that homozygous *TBXT* mutations could cause early fetal death in mice whereas mice with heterozygous *TBXT* mutations were short-tailed; this provided proof that this trait was linked to the *TBXT* gene. However, the embryonic development of brachyury, an encoded protein, also plays an extremely important role [21]. Brachyury, an important nuclear transcription factor throughout embryonic development [22], participates in and regulates numerous signaling pathways that influence critical processes such as mesoderm differentiation and somite segmentation [23,24]. Nucleotides 333 and 334 in sheep *TBXT* correspond with residues 111 and 112 in the T-box domain of the brachyury protein. Interestingly, most mice and cats [25,26] with the short-tailed phenotype are heterozygous for *TBXT* mutations and homozygous *TBXT* mutations have a negative influence on early embryonic development. In a 2013 study of Manx cats with a short-tailed phenotype [26], the researchers found that a heterozygous mutation in *TBXT* could cause a phenomenon known as haploinsufficiency. When one allele is mutated, the other allele can still be normally expressed but in cases of haploinsufficiency, having only half of the usual level of protein is not sufficient to maintain normal cell function; this is the phenomenon responsible for Manx cats having a short-tailed phenotype. The same hypothesis was proposed in a 2016 study [27] of cats with the short-tailed phenotype in Southeast Asia.

Hulunbuir sheep are a unique breed of Mongolian sheep in the Xini River Basin in the Hulunbuir region of the Inner Mongolia Autonomous Region of China [19,28], which can be divided according to the two main strains [29]: Barag sheep with the fat-long-tail phenotype; and short-tailed sheep with the short-tail phenotype. The very short tails of short-tailed sheep do not cover the anus whereas Barag sheep have tails that reach to their hocks or lower. With the exception of the tail phenotype, Barag sheep and short-tailed sheep are otherwise nearly identical [19], making these two sheep strains with different tail morphologies good biological models to research the mechanisms underlying the formation of short tails.

In the study of Zhi [19], the results of genome resequencing suggested that mutations in two linked nucleotides in *TBXT*, 333 and 334, might affect the number of caudal vertebrae in sheep. In the hybridization experiments of Han [18], several sheep with the short-tailed phenotype were found to have heterozygous mutations and individuals with heterozygous *TBXT* mutations could retain the short-tailed phenotype. This was different from the discoveries in mice and other mammals. *TBXT* homozygous mutations are not lethal during the embryonic development of short-tailed sheep. This suggests that the mechanism by which the *TBXT* mutation causes the short-tailed phenotype in sheep is different from that in mice and cats. Due to the widespread breeding of Barag sheep and short-tailed sheep in the Hulunbuir area, it is not uncommon for the offspring to be short-tailed with *TBXT* heterozygosity.

Farmers require a fast and low-cost genotyping method to ensure the breeding of homozygotes and to avoid progeny trait segregation during short-tailed sheep breeding, thereby reducing the time it takes to detect the genotype of progeny [30]. It can be difficult for farmers raising sheep with a short-tailed phenotype to distinguish between homozygotes and heterozygotes for the *TBXT* mutation. High-resolution melting (HRM) has been used to genotype various diseases and economic traits [31,32,33] because it is less expensive and time-consuming than Sanger sequencing and the TaqMan method [34]. The basic principle of HRM is that the heterozygous sample is amplified by PCR to generate DNA fragments and a mixture of two types of homoduplex DNA and two types of heteroduplex DNA will form upon the denaturation and renaturation of these fragments. A unique melting curve is formed during the melting of the resulting DNA fragments that can be distinguished from those of the homozygous DNA fragments [35]. Many mutated genes for economic traits in sheep and cattle have been identified by HRM analyses. These mutated genes include bone morphogenetic protein 15 (*BMP15*) [30], growth differentiation factor 9 (*GDF9*) [36], and major histocompatibility complex class II DRB3 (*BoLA-DRB3*) [37]. These well-established assay protocols demonstrate the benefits and viability of applying an HRM analysis in sheep breeding. In our research, we propose an HRM analysis using unlabeled probes and asymmetric PCR. The unlabeled probes ensure that the Tm difference between the homozygous and heterozygous samples is 0.3 °C, which allows the two samples to be clearly distinguished. The HRM method can be used to effectively select homozygous sheep, thus helping farmers to produce more sheep with the short-tailed phenotype.

## 2. Materials and Methods

### 2.1. Ethical Statement

All sheep experimental procedures and protocols were approved and authorized by the animal care and use committee of the Inner Mongolia Agricultural University (Inner Mongolia Autonomous Region, China) in this study (License No. 2020008).

### 2.2. Materials

Short-tailed sheep and Barag sheep were grown in Ewenki Autonomous County (Inner Mongolia Autonomous Region, China). We randomly chose 60 short-tailed sheep and 60 Barag sheep of the same age from 3 farms (40 on each farm). An equal number of rams and ewes was used to eliminate the effect of sex. A 2 mL sample of venous blood from each sheep was collected in a vacuum container containing EDTA and stored at −20 °C. Among the selected sheep flocks, 16 Barag sheep and 14 short-tailed sheep with known genotypes were selected to obtain caudal vertebrae X-ray images.

### 2.3. Methods

#### 2.3.1. Genomic DNA Extraction

Genomic DNA was extracted using a FastPure Blood DNA Isolation Reagent kit (Vazyme, Nanjing, China) according to the manufacturer’s instructions. DNA concentration and purity were evaluated using a NanoDrop^TM^ 2000 ultraviolet-visible spectrophotometer (Thermo Fisher Scientific, Waltham, MA, USA). The extracted DNA was diluted to roughly 50 to 70 ng/μL.

#### 2.3.2. PCR Amplification and Sequence Alignment

The primers and probes for the HRM and Sanger sequencing analyses were designed using Primer Express 3.0.1 software (Thermo Fisher Scientific, Waltham, MA, USA) (Table 1). The 3′ end of the probe was blocked with a C3 spacer and the primers and probes were synthesized by Sangon Biotech Co. Ltd. (Shanghai, China). A total reaction volume of 50 μL was used for the PCR amplification procedure, which included 25 μL of 2 × Phanta Max Master Mix (Vazyme, Nanjing, China), 2 μL each of upstream and downstream primers, 20 μL ddH_2_O, and 1 μL of a DNA isolate (concentration ranging from 50 to 70 ng/μL). The PCR reaction program was as follows: pre-denaturation at 95 °C for 3 min (1 cycle); 95 °C for 15 s, 60 °C for 15 s, and 72 °C for 3 s (35 cycles); and an extension at 72 °C for 3 min (1 cycle). The PCR products were purified using an EZ-10 Column DNA Purification kit (Sangon Biotech, Shanghai, China) following the manufacturer’s instructions, and the purified DNA fragments were sent to Sangon Biotech Co. Ltd. (Shanghai, China) for Sanger sequencing. The amplified DNA fragments were aligned with the sheep *TBXT* reference sequence (NCBI accession number: 101114280) using Chromas 2.6.5 software (Technelysium Pty Ltd., SouthBrisbane, QLD, Australia).

#### 2.3.3. HRM Reaction

Using an HRM analysis kit (Tiangen, Beijing, China), the total reaction volume was 10 μL, including 5 μL of 2 × HRM Analysis PreMix (with EvaGreen) with 0.2 μL each of upstream and downstream primers, 10 μM of downstream primers, and 1 μM of upstream primers as well as 0.2 μL of an unlabeled probe (concentration 10 μM), 3.9 μL of ddH_2_O, and 0.5 μL of DNA amplification using PikoReal^TM^ real-time fluorescence quantitative PCR equipment (Thermo Fisher Scientific, Waltham, MA, USA). The PCR reaction program included pre-denaturation at 95 °C for 2 min and 50 cycles at 95 °C for 10 s and 60 °C for 30 s. The subsequent melting curve analysis consisted of 2 steps: denaturation at 95 °C for 30 s and renaturation at 60 °C for 1 min followed by a continuous fluorescence reading mode from 60 to 95 °C with a ramp rate of 0.02 °C/s. To ensure reliable genotyping results, the reactions were performed in triplicate. PikoReal^TM^ 2.2.250.602 software (Thermo Fisher Scientific, Waltham, MA, USA) was used to evaluate the melting curves, normalize the plots of fluorescence versus temperature, and conduct automated genotyping based on the analysis of the HRM characteristics.

#### 2.3.4. Acquisition of X-ray Images of Sheep Caudal Vertebrae

To observe the number of caudal vertebrae in the Barag sheep and short-tailed sheep, we obtained X-ray images of the caudal vertebrae of sheep of a known genotype using a portable X-ray machine (General Electric Company, Fairfield, CT, USA).

#### 2.3.5. Statistical Analysis

All statistical analyses were performed using SPSS.25 software (IBM Corp., Armonk, NY, USA). Chi-squared tests were used to investigate the genotypic differences between Barag and short-tailed sheep as well as the differences in the number of caudal vertebrae between the homozygous and heterozygous short-tailed sheep.

## 3. Results

**Figure 1:**
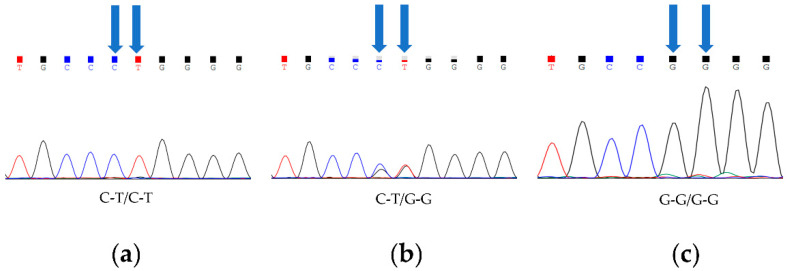
Three genotypes of *TBXT* in Barag sheep and short-tailed sheep. Blue arrows point to two polymorphic sites. (**a**) Fragments of chromatograms with genotype C-T/C-T. (**b**) Fragments of chromatograms for heterozygous genotype C-T/G-G. (**c**) Fragments of chromatograms with genotype G-G/G-G.

A statistical analysis and a chi-squared analysis were performed on the Sanger sequencing results of 60 Barag sheep and 60 short-tailed sheep (Table 2). In both groups of sheep, we detected a total of three genotypes (C-T/C-T, C-T/G-G, and G-G/G-G; Figure 1). In the Barag sheep, nucleotides 333 and 334 in *TBXT* were both G. No individuals with c.333G > C or c.334G > T were found. We discovered that the primary genotype in the short-tailed sheep population was c.333G > C; c.334G > T homozygotes (C-T/C-T) accounted for 86.7% of the sheep and the heterozygous genotype (C-T/G-G) accounted for 13.3% of the sheep. Similarly, we found no individuals with G at *TBXT* nucleotides 333 and 334 among the short-tailed sheep. This result showed that *TBXT* (c.333G > C or c.334G > T) was strongly associated with the sheep tail traits (*p* < 0.05) (Table 2).

**Figure 2:**
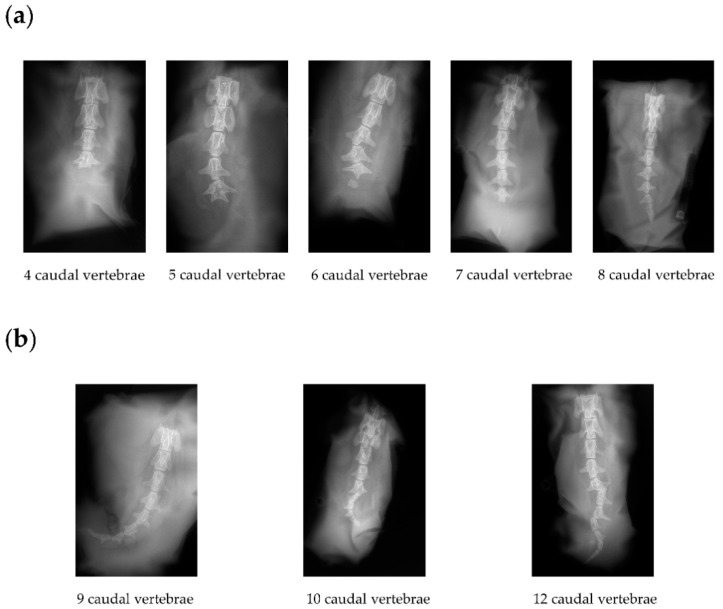
X-ray images of the caudal vertebrae of Barag sheep and short-tailed sheep. (**a**) Representative X-ray images of the caudal vertebrae of short-tailed sheep. (**b**) Representative X-ray images of the three types of caudal vertebrae of Barag sheep.

To investigate if the homozygous short-tailed sheep (C-T/C-T) had fewer or shorter caudal vertebrae than the heterozygous short-tailed sheep (C-T/G-G), we obtained X-ray image phenotypes of 14 short-tailed sheep and 16 Barag sheep. In Figure 2, we show the representative X-ray images of the caudal vertebrae of the short-tailed sheep and Barag sheep. It is worth noting that the ends of the caudal vertebrae of the short-tailed sheep were deformed. The number of caudal vertebrae of the Barag sheep were 9, 10, or 12 (Figure 2b and Table 3) whereas the number of caudal vertebrae in the short-tailed sheep ranged from 4 to 8 (Figure 2a and Table 3). We recorded the number of caudal vertebrae corresponding with the two genotypes of short-tailed sheep and performed a chi-squared test (Table 4). The results showed that the genotype of short-tailed sheep was not associated with the number of caudal vertebrae (*p* > 0.05). This showed that it is difficult to distinguish homozygous individuals from heterozygous individuals by observing the tail phenotype alone.

**Figure 3:**
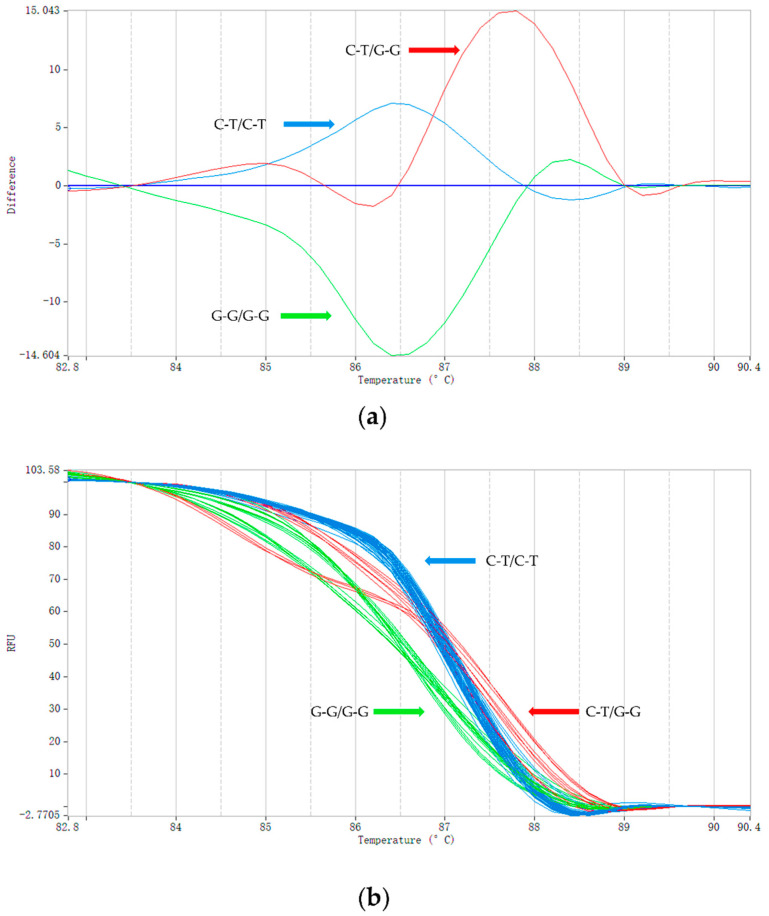
HRM genotyping results. (**a**) Typical shapes of the melting curves corresponding with the three genotypes (C-T/C-T, C-T/G-G, and G-G/G-G). The color of the curve is automatically assigned by PikoReal^TM^ software. The vertical axis represents the change in fluorescence intensity and the abscissa represents the Tm value. The value of the abscissa corresponding with the main peak of each curve is the Tm value of the amplified fragment. (**b**) The melting curve image generated during the genotyping of Barag sheep and short-tailed sheep (a total of 32 samples are shown in the image; each sample was run in triplicate).

Using the HRM method, we genotyped 60 short-tailed sheep and 60 Barag sheep (Figure 3). We added unlabeled probes complementary to the detected DNA fragments using asymmetric PCR to form a single strand of nucleotides [38]. During the PCR amplification, the unlabeled probes competed with and bound to the template DNA sequence of the genotype G-G/G-G. Therefore, the Tm of sample genotype G-G/G-G was the lowest at only 86.84 °C and that of the heterozygous sample C-T/G-G, for which only half the sequence could be combined with the unlabeled probe, was 87.32 °C. The homozygous genotype of the short-tailed sheep, C-T/C-T, did not bind to the unlabeled probes so the Tm was 87.63 °C, which was the highest. The melting curves of the three genotypes (C-T/C-T, C-T/G-G, and G-G/G-G) can be clearly seen in Figure 3. HRM using unlabeled probes and asymmetric PCR increased the Tm difference between the homozygotes and heterozygotes compared with HRM; with smaller amplicons, the accuracy of the genotyping was improved [38,39,40]. To ensure that the HRM genotyping results were accurate, we compared them with the results of Sanger sequencing, which demonstrated that the same genotype was obtained using both methods.

## 4. Discussion

One of the purposes of the tail of a sheep is to help the sheep retain energy and fat [41]. When food is scarce, fat from the tail can be utilized to keep the body functioning smoothly [14]. Tail docking not only causes a stress reaction in lambs but also increases the risk of wound infection [42,43,44]. Short-tailed sheep can be actively raised by farmers to avoid the previously mentioned aberrant state and enhance animal welfare [9,45,46,47]. Compared with the danger of tail docking [13], producing sheep with a short-tailed phenotype in their natural condition is more in line with current sheep breeding demands. The number and length of caudal vertebrae determine the overall length of the tail and although short-tailed sheep do not have the same amount of fat in their tails as long-tailed sheep, their bodies compensate by increasing intramuscular fat [48] because tail fat can only be deposited on the bones of the caudal vertebrae [9]. The flesh quality of short-tailed lambs is superior to that of long-tailed lambs. Two linked mutations in the *TBXT* gene, c.333G > C and c.334G > T, have been linked to the short-tailed phenotype in various animals and can be utilized as genetic markers in breeding operations. As a result, utilizing *TBXT* (c.333G > C or c.334G > T) for homozygous short-tailed sheep breeding is reliable [9,16,17].

Unlike other genotyping studies [49], most of the mutation sites identified by HRM involve only one base pair. However, there are two linked mutation sites to be identified in sheep with the short-tailed phenotype. The Tm generated by two linked mutations cannot be directly increased by HRM utilizing short amplicons. To improve the Tm difference between the homozygous and heterozygous DNA fragments in this experiment, we used unlabeled probes and asymmetric PCR. As a result, the detected fluorescent signal was enhanced. In screening individuals with genetic mutations in flocks, an HRM analysis has a wide range of applications [35,50]. This technology is less costly than TaqMan and Sanger sequencing but has the same precision and applicability. This approach primarily employs nonspecific binding of saturated fluorescent dyes to double-stranded DNA during PCR [51]. Fluorescent colors are removed from the DNA fragments when they are heated and denatured. Using a fluorescent trap, the binding of double-stranded DNA and fluorescent dyes can be observed in real-time during the heating phase [52]. The melting curve is the result of the detection machine monitoring the fluorescent signal in real-time as it changes. When the bases in the amplified fragments are different, the Tm values of the DNA fragments also differ and distinct melting curves can be used to distinguish different genotypes.

Utilizing unlabeled probes and asymmetric PCR in the HRM system can increase Tm differences across the genotypes [31,53]; therefore, we recommend that this method is used because of the small difference in Tm between the homozygotes and heterozygotes for the short-tailed sheep *TBXT* mutation. When designing unlabeled probes for HRM, we discovered that the accuracy of genotyping could be improved by placing the mutation in the middle of the probe fragment, which increased the sensitivity of detecting both homozygous and heterozygous individuals. Furthermore, we discovered that the concentration of the DNA template in the HRM system should be investigated. According to previous studies [54], the DNA template concentration is an important determinant for the accuracy of genotyping results; however, when the DNA concentration was less than 5 ng/μL in our tests, the genotyping results were inaccurate because multiple nonspecific amplifications occurred during the HRM reaction, reducing the PCR amplification efficiency. The application of molecular identification methods can facilitate a more rapid and accurate determination than a phenotypic identification; therefore, it is important to establish a rapid detection technology for economically important traits in sheep.

## 5. Conclusions

In conclusion, we developed an HRM approach based on unlabeled probes and asymmetric PCR that could reliably detect *TBXT* heterozygotes in short-tailed sheep. This method may be used in short-tailed sheep breeding.

## 6. Patents

An application for a Chinese invention patent has been submitted for this work (application number: 202110056027.2)

## Figures and Tables

**Table 1:** Primers used in HRM and Sanger sequencing.

Name	Sequence (5′-3′)	Analysis Type	PCR Product Length
*TBXT*-F	TGCGCCCCTTCCTTTTCAG	HRM/PCR	203 bp
*TBXT*-R	GGGGGAGTCGGGGTGGATGTAG	HRM/PCR	
*TBXT*-Probe	GCTTGCCCCAGGGCACCCA	HRM	

**Table 2:** Genotyping statistics for Barag sheep and short-tailed sheep.

Breed	Genotype (*TBXT* c.333G > C; c.334G > T)			X^2^	*p*-Value
	G-G/G-G	C-T/G-G	C-T/C-T		
Barag sheep	100%	0	0	154.87	2.07 × 10^−35^
Short-tailed sheep	0	13.3%	86.7%		

**Table 3:** Statistics on the number of caudal vertebrae in Barag sheep and short-tailed sheep.

	Number of Caudal Vertebrae									Total
	4	5	6	7	8	9	10	11	12	
Number of short-tailed sheep	2	4	1	6	1	0	0	0	0	14
Number of Barag sheep	0	0	0	0	0	6	8	0	2	16

**Table 4:** Statistics on the number and genotype of caudal vertebrae in short-tailed sheep.

	Number of Caudal Vertebrae					Total	X^2^	*p*-Value
	4	5	6	7	8			
Number of homozygotes	1	2	1	3	0	7	2.282045	1
Number of heterozygotes	1	2	0	3	1	7		

## Data Availability

No new data were created or analyzed in this study. Data sharing is not applicable to this article.
